# CCL5 as a Prognostic Marker for Survival and an Indicator for Immune Checkpoint Therapies in Small Cell Lung Cancer

**DOI:** 10.3389/fmed.2022.834725

**Published:** 2022-02-17

**Authors:** Yichun Tang, Yueyang Hu, Yuchun Niu, Lei Sun, Linlang Guo

**Affiliations:** ^1^Department of Pathology, Zhujiang Hospital, Southern Medical University, Guangzhou, China; ^2^Department of Hepatobiliary Surgery, Zhujiang Hospital, Southern Medical University, Guangzhou, China

**Keywords:** CCL5, tumor microenvironment, CIBERSORT, tumor infiltrating immune cells, small cell lung cancer (SCLC)

## Abstract

The standard treatment for small cell lung cancer (SCLC) has not changed in decades. Recently, important advances have been made in immunotherapy. However, analysis of these trials suggests that only a small proportion of patients benefit from immune checkpoint blockade (ICB). Identifying these patients is a clinical challenge. In this study, we applied the ESTIMATE calculation to calculate immune scores in 159 cases of SCLC from two published cohorts. COX regression analysis was used to analyze the differentially expressed genes (DEGs) with high and low immune score. We found that CCL5 expression was positively correlated with survival in SCLC patients. In addition, we verified the effect of CCL5 on survival and response to treatment in another cohort that received immunotherapy. Meanwhile, Gene set enrichment analysis (GSEA) showed that genes with high expression of CCL5 were mainly enriched in immune-related activities. The result of Tumor Immune Dysfunction and Exclusion (TIDE) demonstrated that CCL5 was a potential biomarker to predict response to ICB for SCLC, which is correspondent with the result in verified cohort. These results suggest that CCL5 may be the reason for TME to maintain its immune dominance, making it a favorable factor for ICB. Therefore, CCL5 levels may help to outline the prognosis of patients with SCLC.

## Introduction

Small cell lung cancer (SCLC), which accounts for 15% of all lung cancers, is a highly malignant neuroendocrine tumor (1). At present, the treatment of small cell lung cancer is limited. Surgery, platinum-containing chemotherapy and radiotherapy remain the main treatments (2, 3). SCLC responds well to chemotherapy, whereas resistance often develops rapidly after a brief remission period.

Immunotherapy refers to the use of tumor cell immunogenicity to stimulate the host to kill the tumor cells. At present, CTLA-4 and PD-1/ PD-L1 are the most popular immunotherapy targets. Immunotherapy has been approved as a second-line regimen of SCLC according to CheckMate032 and KEYNOTE-028/158 by Food and Drug Administration (FDA) (4, 5).It has also shown encouraging results in small cell lung cancer (4, 6). In IMpower-133, patients treated with etoposide/carboplatin/atezolizumab had longer clinical survival than the control group as a first-line regime (7). However, patients with SCLC benefit much less from immunotherapy than patients with non-small cell lung cancer. SCLC tumors exhibit fewer immune cells in the tumor immune microenvironment (TIME), which may account for poor response to immune checkpoint blocking (8).Molecular markers that determine prognosis and the efficacy of immunotherapy have not been identified thus far. Meanwhile, a highly variable proportion of PD-L1 protein expression has been found in SCLC (9, 10). Unfortunately, both IMpower133, CASPIAN study and CheckMate032 study showed that there was no correlation between PD-L1 expression level and therapeutic effect of experimental groups (4, 11).

Tumor mutation burden (TMB) refers to the number of substitutions, insertions and deletions per megabyte of the exon coding region of the evaluated gene. Genomic analysis of SCLC has identified two defective tumor suppressor genes (p53 and RB1) that cause genomic instability (12). Thus, SCLC is characterized by a high mutation load, which is theoretically suitable for immunotherapy (12, 13). In Checkmate 032, tumor mutation burden was higher among patients with response to either monotherapy or combination therapy, which indicates that tumor mutation burden has prognostic value (14). But it requires more data to prove that.

In this study, aiming to discover molecular markers that play a key role in prognosis and the efficacy of immunotherapy for SCLC patients, we used ESTIMATE algorithm to calculate immune scores in SCLC samples from 159 cases of SCLC from two published cohorts and used Cox regression analysis to search for prognostic immune markers, leading to the identification of C-C Motif Chemokine Ligand 5 (CCL5). To further elucidate the potential effect of CCL5 in SCLC, we carry out the gene co-expression network analysis, CIBERSORT algorithm for estimations of the proportion of immune cell infiltrate, Tumor Immune Dysfunction and Exclusion (TIDE) algorithm for prediction of response to immune checkpoint blockade and Gene Set Enrichment Analysis. These findings may make a meaningful contribution to the development of immune therapy for SCLC patients.

## Materials and Methods

### Acquisition of Data

Transcriptome RNA-seq data and clinical records of SCLC patients were obtained from the supplementary file of the studies reported by George et al. (12), Jiang et al. (15), and Roper et al. (16).

### ImmuneScore Calculation

The infiltration level of immune cells was inferred from gene expression data in the studies by George and Jiang by calculating the ImmuneScore derived from the ESTIMATE algorithm using the estimate package in R (version 4.0.5). A higher ImmuneScore indicated a greater ratio of immune cell infiltrate in the TME.

### Identification of DEGs Between High and Low ImmuneScore Groups

Eighty-one tumor samples in the George study and 78 tumor samples in the Jiang study were classified into the high score group or low score group based on the comparison to the median score of the ImmuneScore, respectively. The limma package in R was used to perform differential analysis of the gene expression of high score group and low score group samples. Genes with LogFC > 1.0 and false discovery rate (FDR) <0.05 were identified as DEGs. Volcano plots and heatmaps were produced by the ggplot2 and pheatmap packages in R, respectively.

### Survival Analysis

The survival package in R were applied for the survival analysis. Seventy-five cases in the George study and 48 cases in the Jiang study with survival data were used for survival analysis. Seventeen cases in Roper study were used as an independent external verification cohort. The optimal cutoff point for the expression of CCL5 was determined by the “surv_cutpoint” algorithm. Kaplan–Meier (KM) analysis was performed to compare the survival outcomes of the CCL5 low and high expression groups, and log rank was used as the statistical significance test.

### COX Regression Analysis

The survival package in R was used for univariate Cox regression analysis. The expression levels of the DEGs were analyzed using a univariate Cox model, and the top 15 genes ordered by *p*-value from small to large in univariate Cox are shown in the forest plot. CCL5 expression levels and all clinical factors in the Jiang study and Roper study were analyzed using a univariate Cox model and multivariate Cox regression model, and factors with *P* < 0.05 in the multivariate Cox regression model were identified as independent prognostic factors.

### Gene Co-expression Network Analysis

Seventy-eight samples from Jiang's study were used to conduct a co-expression analysis. The correlation between the CCL5 expression level and other DEGs was calculated using R. Genes with a *P* < 0.05 were considered significant. These genes and Pearson's correlation coefficients were uploaded to Cytoscape software (http://www.cytoscape.org) (version 3.8.1) to map gene co-expression networks.

### Gene Ontology and Kyoto Encyclopedia of Genes and Genomes Enrichment Analysis

A total of 412 DEGs were used for GO and KEGG enrichment analyses, which were performed with the clusterProfiler and ggplot2 packages in R. Terms with both p- and q-values < 0.05 were considered significantly enriched.

### Estimations of the Proportion of Immune Cell Infiltrate

The CIBERSORT algorithm in R was applied to estimate the proportion of 22 types of immune cells that had infiltrated tumor samples in the studies by George and Jiang. Samples with CIBERSORT *p* < 0.05 were selected for subsequent analysis. The overall infiltration of 22 types of immune cells in all selected samples is shown in the histogram, and the correlation between immune cells is shown in the heatmap.

### Correlation Analysis of the Immune Microenvironment

The correlation between CCL5 and each type of immune cell expression level was calculated by R, and scatter plots and fitted regression lines were drawn through the ggplot2 package in R. Differential analysis for infiltrating immune cells and immune checkpoints was performed in the CCL5 low and high expression group, and the vioplot and ggpubr packages in R were used for plotting to display the outcomes of analysis.

### Prediction of Response to Immune Checkpoint Blockade and Validation

Tumor Immune Dysfunction and Exclusion (TIDE, http://tide.dfci.harvard.edu/) is a computational framework developed to evaluate the potential of tumor immune escape from the gene expression profiles of cancer samples. The TIDE score computed for each tumor samples can serve as a surrogate biomarker to predict response to immune checkpoint blockade, the higher the TIDE score means the lower the possibility of response to immune checkpoint blockade. Expression data in Jiang study was uploaded for the prediction. The responding situations of durvalumab treatment in Roper cohort were used for validation. The receiver operating characteristic (ROC) curve was used to evaluate the response to ICB of CCL5. A *p* < 0.05 was considered statistically significant.

### Gene Set Enrichment Analysis

Gene set enrichment analysis (GSEA) was performed using GSEA-4.1 software to elucidate the molecular mechanisms of CCL5. The gene sets used included the Hallmark gene sets, C2 sets (curated gene sets), and C7 gene sets (immunologic signature gene sets). The C2 collection is divided into the following two sub-collections: Chemical and genetic perturbations (CGP) and Canonical pathways (CP) sets. Samples of 48 cases from Jiang's study divided into the CCL5 low expression group and high expression group were used for GSEA. Gene sets with |NES| > 1, NOM *p* < 0.05 and FDR q <0.25 were considered significant.

## Results

### Identification of TIME Related Genes

**Figure 1:**
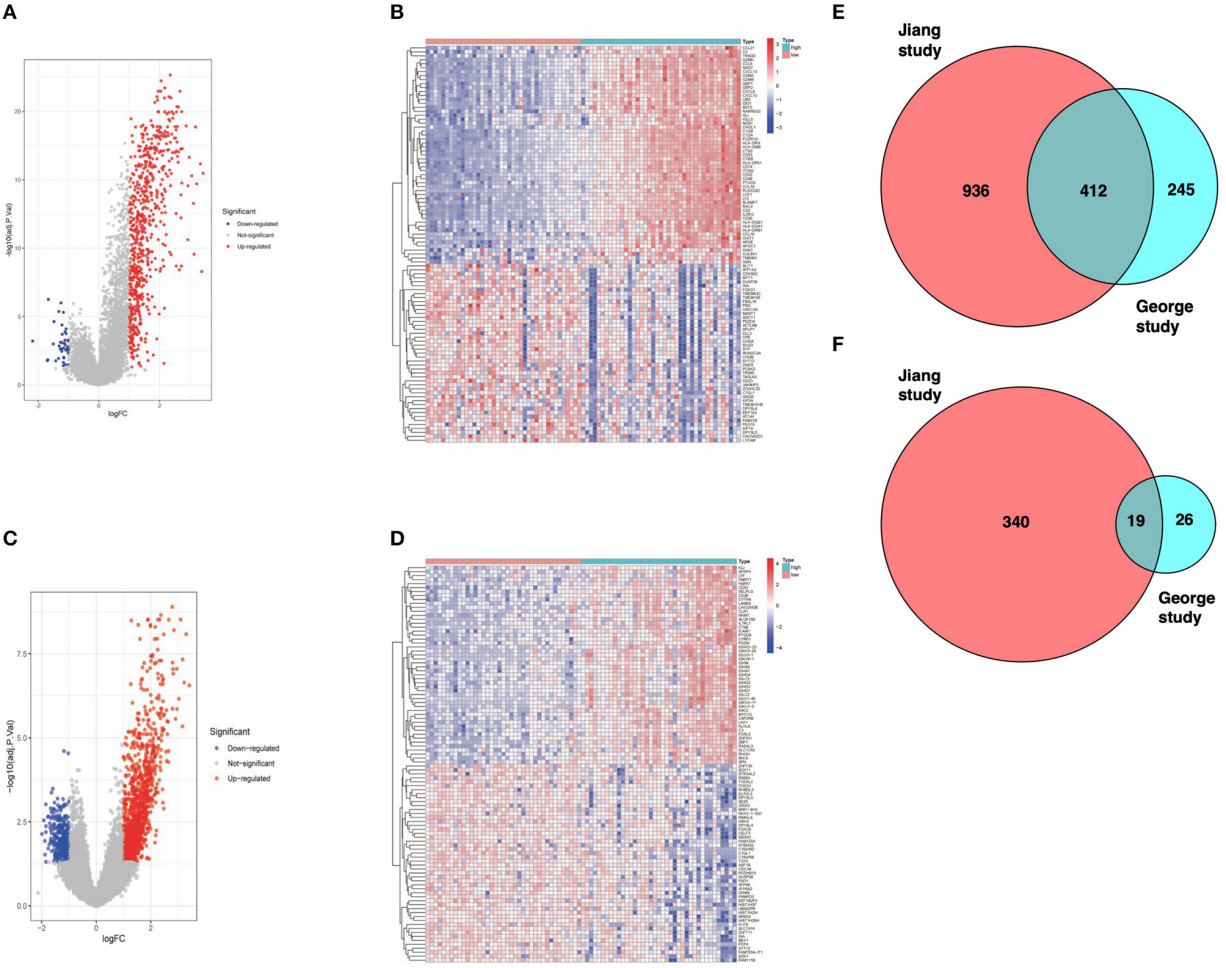
**(A)** Volcano map of dataset from the George cohort (Red dots represent upregulated genes, blue dots represent downregulated genes, and gray dots indicate no difference in expression). **(B)** Heat map of the DEGs identified from the George cohort. **(C)** Volcano map of the Jiang cohort. **(D)** Heat map of DEGs identified from the Jiang cohort. **(E)** Venn diagram. The intersection represents the upregulated genes in the high-score cohort of both datasets. **(F)** Venn diagram. The intersection represents the downregulated genes in the high-score cohort of both datasets.

To assess specific changes in the immune microenvironment of SCLC, we divided patients from the study by George into high and low immunescore groups and found transcriptome differences between the two groups of samples. There were 702 DEGs in the high-score group, including 657 upregulated genes and 45 downregulated genes (Figures 1A,B). Similarly, we used the same algorithm to classify patients from the Jiang cohort and obtained 1707 DEGs, including 1,348 upregulated genes and 359 downregulated genes (Figures 1C,D). Subsequently, we found gene sets that were high or low expression in both groups with high immune scores (Figures 1E,F).

### CCL5 Is a Protective Factor for Prognosis of SCLC

**Figure 2:**
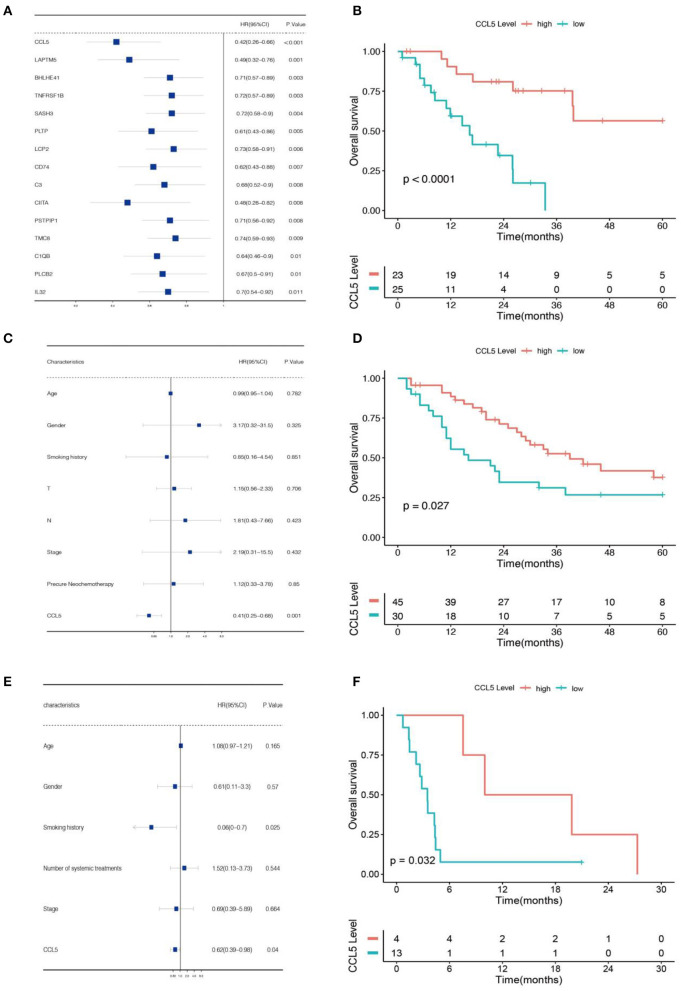
**(A)** Univariate Cox regression analysis of data from the Jiang cohort. **(B)** Association between CCL5 and overall survival based on data from Jiang cohort. **(C)** Multivariate Cox regression analysis of data from the Jiang cohort. **(D)** Association between CCL5 and overall survival based on data from George cohort. **(E)** Multivariate Cox regression analysis of data from the Roper cohort. **(F)** Association between CCL5 and overall survival based on data from Roper cohort.

Then, we used univariate analysis to identify genes associated with prognosis in gene clusters in the Jiang cohort. Among them, the gene with the lowest *P*-value was CCL5 (Figure 2A). Subsequently, we divided SCLC patients from the Jiang and George cohorts into high and low CCL5 expression groups (Supplementary Figures 1A,B). In both cohorts, high CCL5 expression indicates better survival in patients without chromothripsis (Figures 2B,D).

To confirm the relationship between this gene and patients' prognosis, we performed a multivariate analysis. The result demonstrates that CCL5 is an independent protective factor with hazard ratio = 0.41 (Figure 2C; Table 1). Furthermore, we verified this result in Roper cohort, correspondence with the former cohorts, CCL5 indicates better survival in SCLC patients accepting immunotherapy (Figure 2F), as well as an independent protective factor (Figure 2E).

**Table 1:** Univariate Cox model and Multivariate Cox regression model in the Jiang study.

**Clinical characteristic**	**Univariate analysis**			**Multivariate analysis**		
	**HR**	**95%CI**	***p-Value***	**HR**	**95%CI**	***p-Value***
Age	0.99	0.94–1.03	0.545	0.99	0.95–1.04	0.782
Gender	1.02	0.3–3.48	0.974	3.17	0.32–31.5	0.325
Smoking history	0.66	0.26–1.69	0.389	0.85	0.16–4.54	0.851
T	1.61	0.99–2.62	0.056	1.15	0.56–2.33	0.706
N	2.67	1.44–4.96	**0.002**	1.81	0.43–7.66	0.423
Stage	4.21	1.71–10.37	**0.002**	2.19	0.31–15.5	0.432
Precure neochemotherapy	1.07	0.36–3.21	0.902	1.12	0.33–3.78	0.85
CCL5 expression	0.42	0.26–0.66	**0**	0.41	0.25–0.68	**0.001**

*The bold values indicate the statistically significant results*.

### Co-expression Network Analysis of CCL5

**Figure 3:**
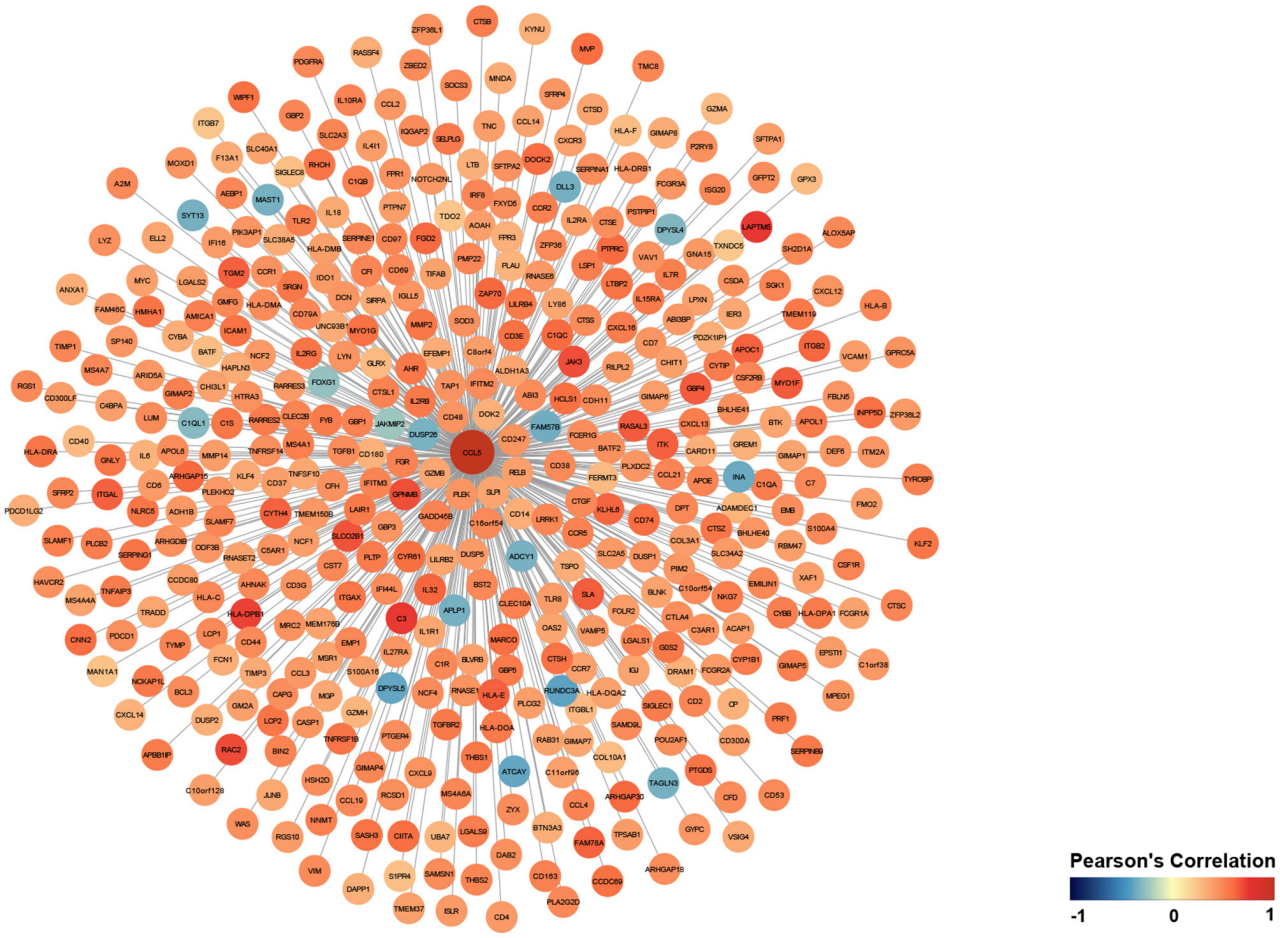
Genes co-expressed with CCL5.

Next, to explore the co-expression genes of CCL5, we calculated the Pearson correlation coefficients between DEGs and CCL5. A total of 427 genes in DEGs with *P* < 0.05 were considered as co-expression genes of CCL5 and visualized via Cytoscape (Figure 3; Supplementary Table 1). We found that LAPTM5, C3 and HLA-DPB1 were the most positively correlated with CCL5, and RUNDC3A, ATCAY and DPYSL5 were the most negatively correlated with CCL5.

### KEGG/GO Biological Process Enrichment for Co-expression Genes of CCL5

**Figure 4:**
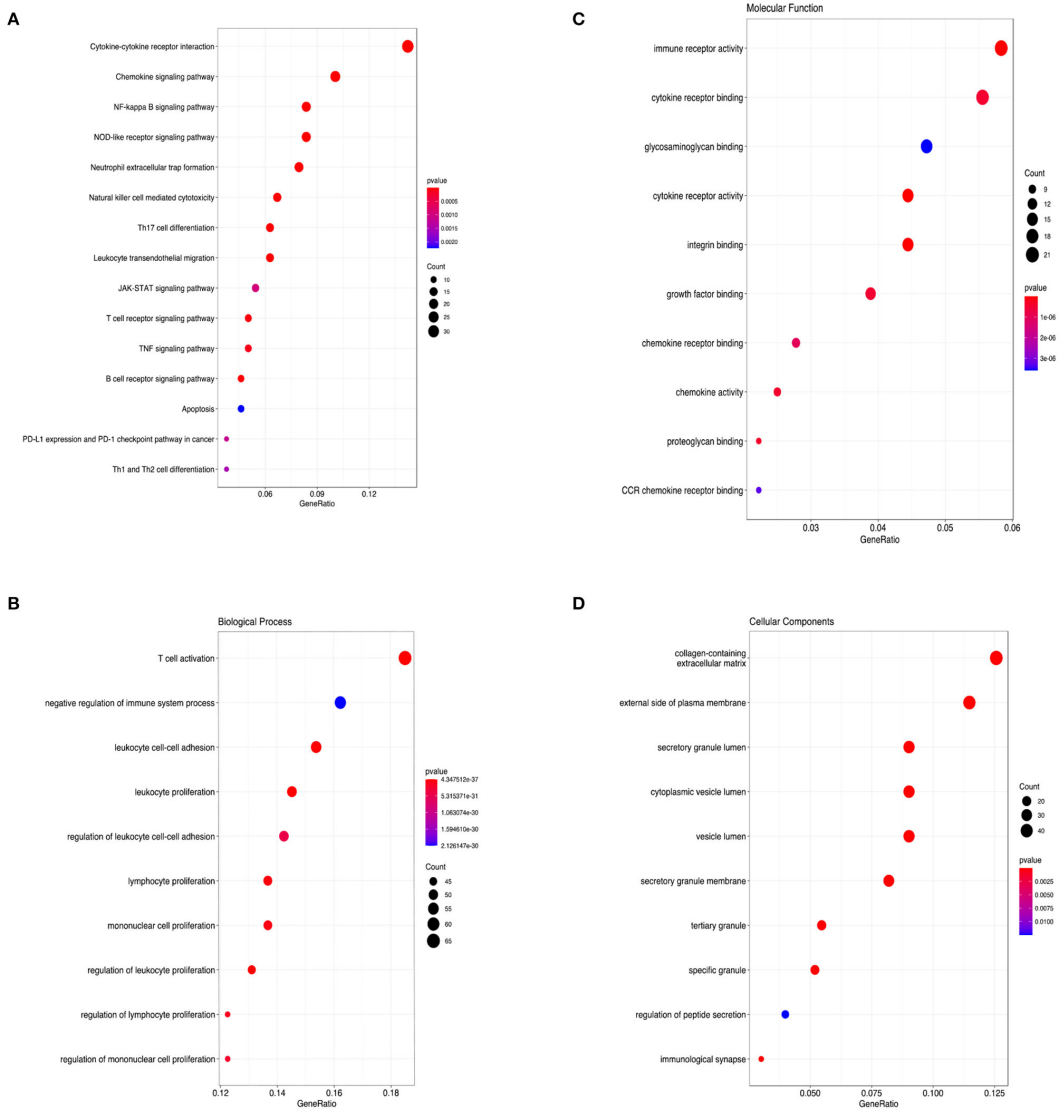
**(A)** KEGG pathway enrichment analysis. **(B)** Biological process enrichment analysis. **(C)** Molecular function enrichment analysis. **(D)** Cellular component enrichment analysis.

KEGG and GO analyses were performed to explore the specific pathways associated with CCL5 and its co-expression genes. The KEGG pathway analysis of CCL5 interactive genes showed that cytokine-cytokine interactions and chemokine signal pathways were the most enriched pathways (Figure 4A) Additionally, GO analysis demonstrated that CCL5 and its co-expression genes were significantly enriched in the T cell activation pathway at biological process (BP) levels (Figure 4B), immune receptor activity at molecular function (MF) levels (Figure 4C) and collagen-containing extracellular matrix at cellular components (CC) levels (Figure 4D). These results suggest that CCL5 is closely related to immune-related molecules.

### Potential Mechanism of CCL5 Regulating the Immune Microenvironment

**Figure 5:**
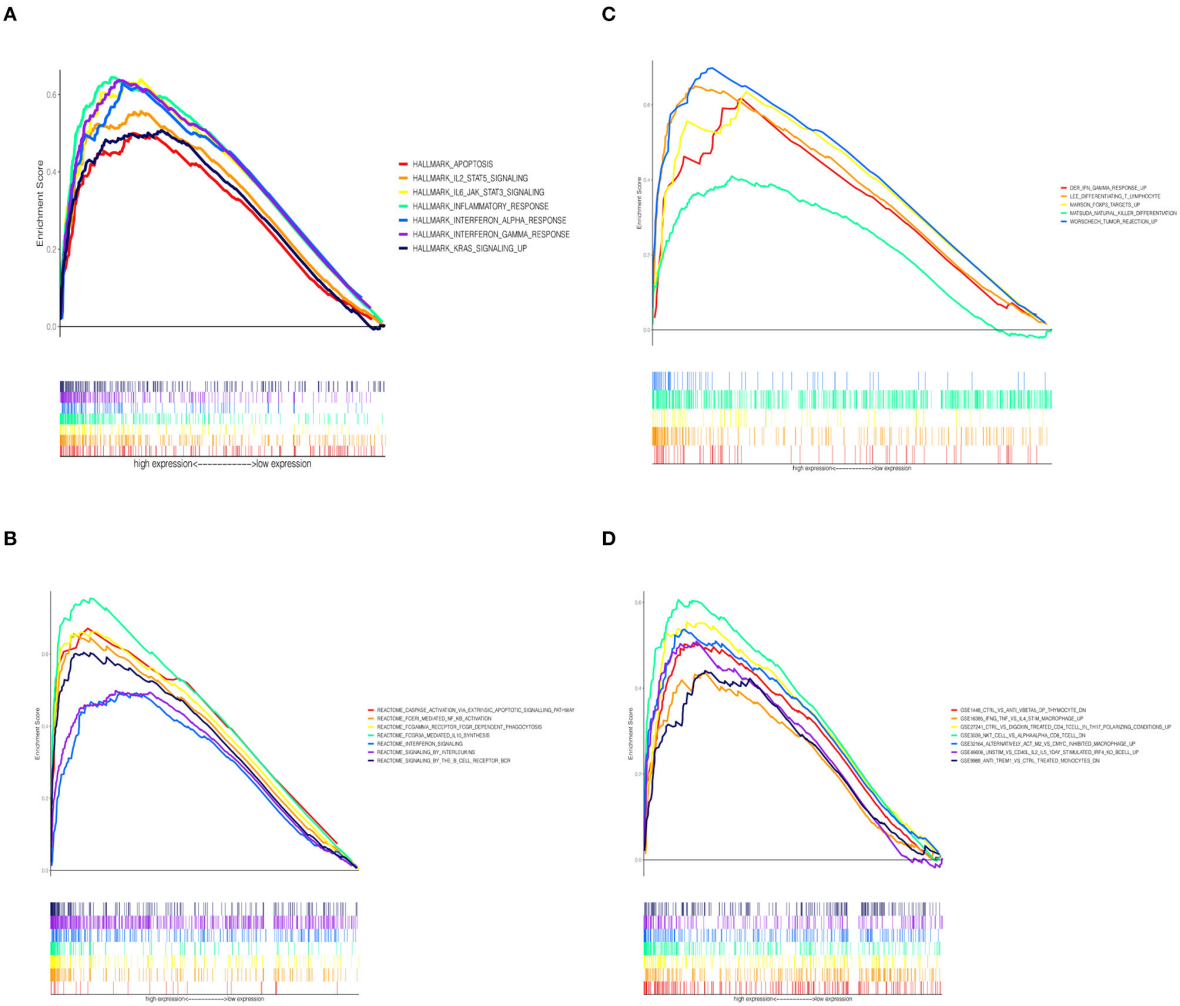
**(A)** Enriched gene sets in the HALLMARK collection in samples with high CCL5 expression. **(B)** Enriched gene sets in REACTOME Pathways databases in samples with high CCL5 expression. **(C)** Enriched gene sets in Canonical Pathways gene sets in samples with high CCL5 expression. **(D)** Enriched gene sets in C7 immune gene sets in samples with high CCL5 expression.

To investigate the correlation between CCL5 expression and immune-related activities, the signaling pathways related to CCL5 expression were studied by GSEA. Tumor samples were divided into high and low groups according to median CCL5 expression levels. The results showed that the hallmark gene set in the CCL5-high expression group was mainly involved in apoptosis and IL2 STAT5 signaling (Figure 5A). In addition, the high CCL5 expression group was enriched in IL-4 signaling pathway of C7 immune gene sets, while enriched in NF-κB activation, FOXP3 target signaling pathways in other gene sets (Figures 5B–D). These results suggest that CCL5 may be an important factor regulating immune-related activities.

### Correlation Between CCL5 and the TICs Proportion

**Figure 6:**
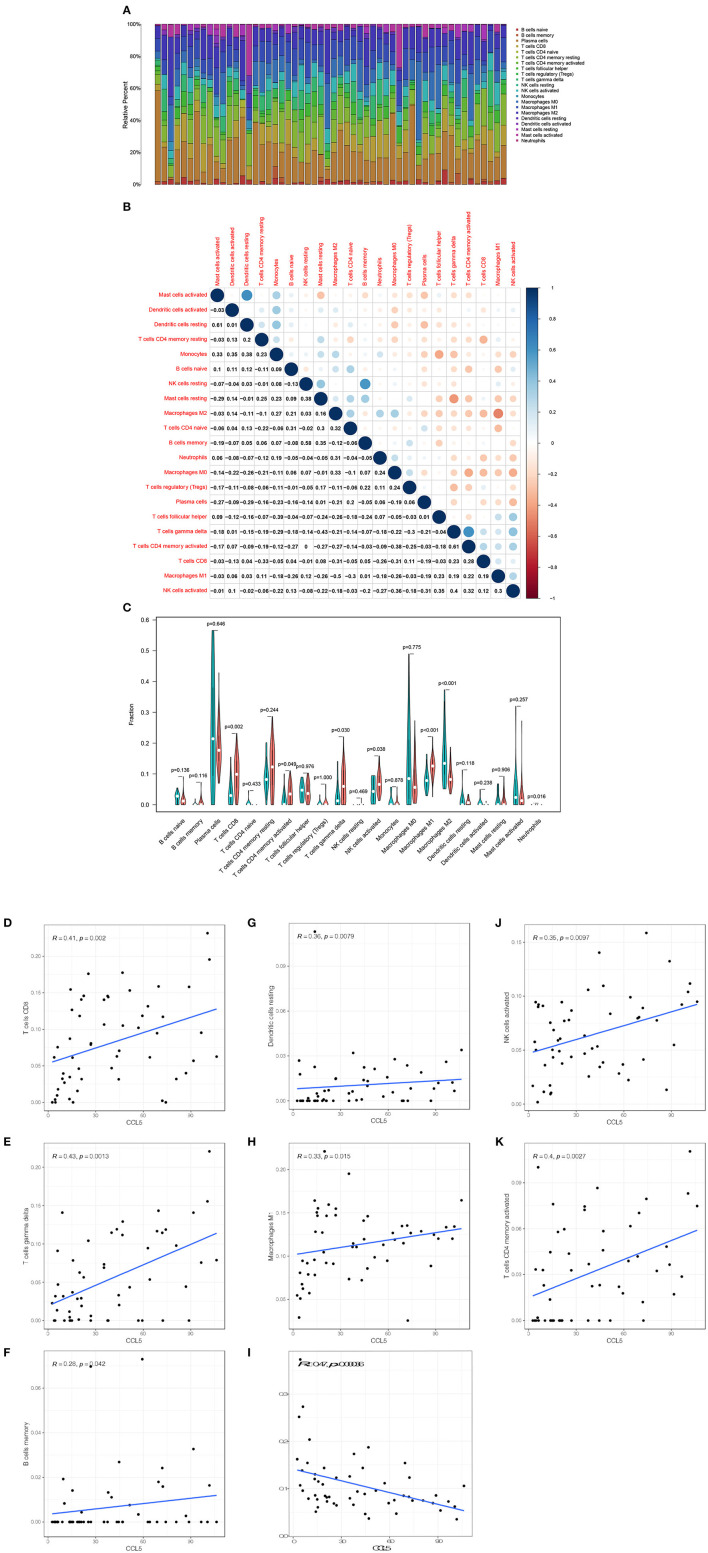
**(A)** Bar plot shows the proportion of 21 types of TICs in SCLC tumor samples. **(B)** The heatmap shows the correlation between 21 TICs and the value in each small box and represents the *P*-value of the correlation between the two cells. The shadows in each tiny color box represent the corresponding correlation values between the two cells, and significance was assessed using Pearson's coefficient. **(C)** The violin plot shows the proportional differentiation of 21 immune cells relative to the median level of CCL5 expression in SCLC tumor samples with low or high CCL5 expression. **(D–K)** Scatter plot showing that the proportion of 8 TICs was correlated with CCL5 expression (*P* < 0.05). The blue line in the figure indicates the fitted line of the linear model, indicating the proportion of immune cells and CCL5 expression. Pearson's coefficient was used for the correlation test.

The CIBERSORT method was used to further confirm the relationship between CCL5 expression and immune components, construct immune cell profiles and analyze the proportion of tumor infiltrating immune subtypes (Figures 6A–C). Eight kinds of TICs were positively correlated with CCL5 expression including CD8+ T cells, gamma delta T cells, CD4+ memory T cells, memory B cells, dendritic cells, M1 macrophages and NK cells whereas M2 macrophages was negatively correlated with CCL5 expression (Figures 6D–K). The above results further confirm that CCL5 expression significantly affects the immune activity in the TIME.

### CCL5 Can Be an Indicator of Efficacy of ICB

**Figure 7:**
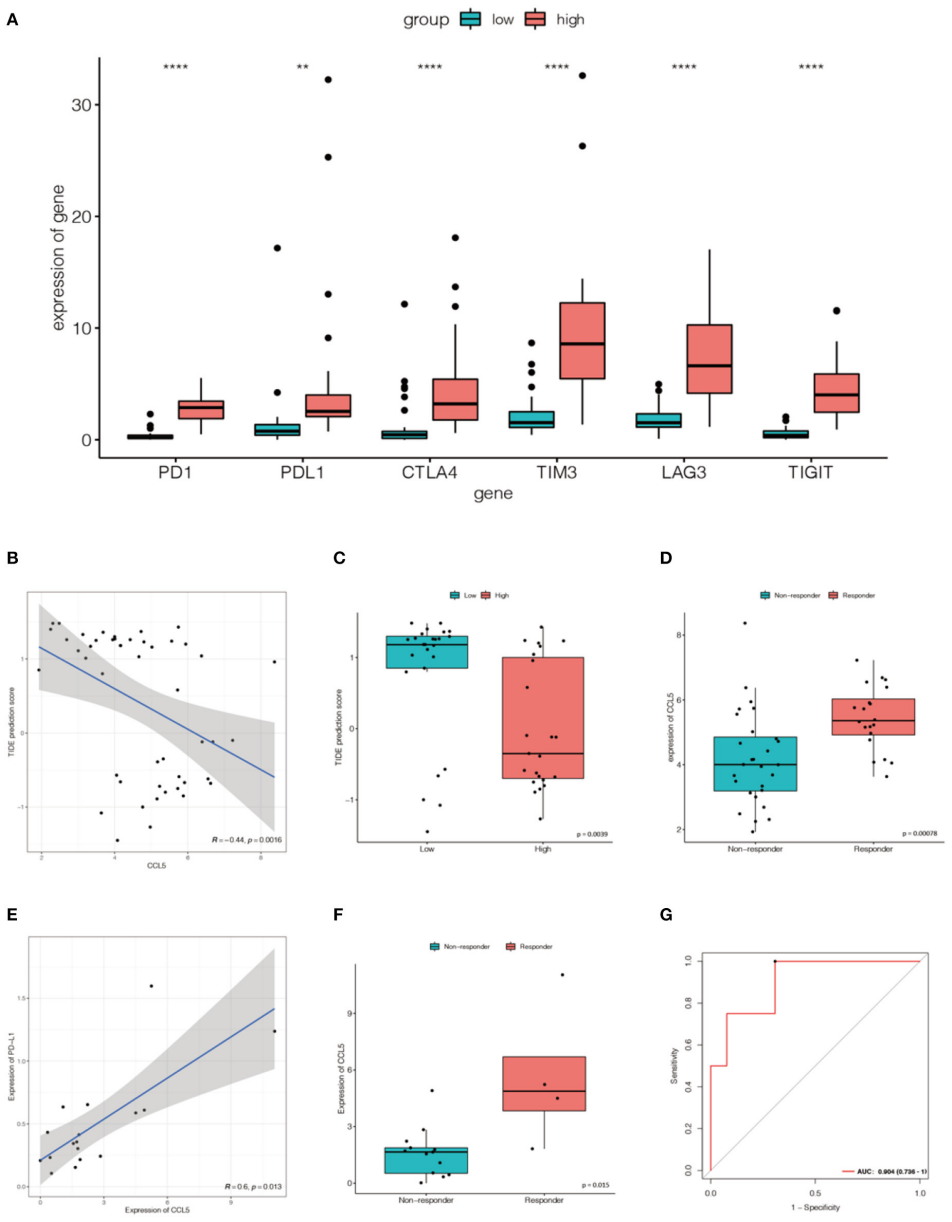
**(A)** The expression of ICPs in the high CCL5 expression group was significantly greater than that in the low CCL5 expression group in the Jiang cohort (**P < 0.001; ****P < 0.0001). **(B)** Relationship between TIDE and CCL5 expression in the Jiang group. **(C)** Distribution of TIDE scores in the high- and low-expression groups. **(D)** CCL5 expression in the Jiang cohort differed between responders and non-responders. **(E)** The expression of CCL5 was positively correlated with that of PD-L1 in Roper's cohort (*p* < 0.05). **(F)** CCL5 expression in the Roper's cohort differed between responders and non-responders. **(G)** Receiver operating characteristic (ROC) curve of CCL5 in Roper's cohort (AUC = 0.904).

To assess the response to immune checkpoint blockade (ICB) based on CCL5 expression, we firstly explored the correlation between CCL5 levels and common immune checkpoints (ICPs). CCL5 expression was associated with ICPs (programmed cell death 1 (PD1), programmed cell death ligand 1 (PD-L1), cytotoxic T lymphocyte antigen 4 (CTLA4), T cell immunoglobulin mucin 3 (TIM-3), lymphocyte activating gene 3 (LAG3), T-cell immune receptors with Ig and ITIM domains (TIGIT), etc.) in the Jiang cohort. ICPs were highly expressed in the group with high CCL5 expression (Figure 7A). The results showed that patients with high CCL5 expression tended to have a high level of ICPs.

Tumor Immune Dysfunction and Rejection (TIDE) is a computational framework used to simulate the two main mechanisms of tumor immune evasion and can provide predictive outcomes of immune checkpoint blockade (17). Elevated TIDE scores may indicate non-response in patients with suppressive T cell infiltration. To better illustrate the predictive power of CCL5 for immunotherapy, TIDE was applied to the Jiang cohort. We were pleasantly surprised to find a negative correlation between TIDE and CCL5 (Figure 7B). In addition, the predicted response suggests that CCL5 may be a good predictor of immune checkpoint blockade for SCLC (Figures 7C,D).

To more credibly illustrate this result, we validated a positive correlation between CCL5 expression and PD-L1 expression in the Roper cohort, in which patients received the anti-PD-L1 antibody durvalumab and poly(adp-ribose) polymerase (PARP) inhibitor Olaparib (Figure 7E). The expression of CCL5 was statistically different between responders and non-responders (Figure 7F). CCL5 was highly expressed in the responders group. Receiver operating characteristic (ROC) curve also reveals that CCL5 is a good marker to predict the result of immunotherapy (AUC = 0.904) (Figure 7G).

## Discussion

The immune landscape of the tumor microenvironment can influence the occurrence, progression, and invasion of cancer, thus influencing patient prognosis. The composition of immune cells in the microenvironment can also predict the efficacy of immunotherapy (18).

CCL5 belongs to the CC motif chemokine family and binds to its receptor CCR5 with high affinity. Different conclusions have been drawn about the role of CCL5 in tumors. Some studies accounted CCL5 for the promotion of tumor development by suppressing the immune response (19), whereas some studies regarded CCL5 as a tumor protective factor associated with high CD8+ T cell infiltration (20, 21). In this study, we first evaluated the relationship between CCL5 and survival in patients with SCLC. We found that high CCL5 expression was associated with longer survival in patients with SCLC. CCL5 is considered the target gene of NF-κB activity, leading to NF-κB activation. These effects ultimately lead to the promotion of T cell-mediated immune surveillance (22, 23). Consistent with this notion, we found that CCL5 was associated with the NF-κB pathway in KEGG enrichment and GSEA analysis. Besides CD8+ T cell, NK cells are emerging as an attractive target for immunotherapy (24, 25). SCLC metastasis is controlled by NK cells (26).NK cells are also a potential therapeutic target for small cell lung cancer (27, 28).

Similarly, the DNA damage response (DDR) inhibition activated the STING/TBK1/IRF3 innate immune pathway, leading to increased levels of chemokines such as CXCL10 and CCL5 that induced activation and function of cytotoxic T lymphocytes (29), while CCL5 recruits T cells in the tumor microenvironment via IFN (11). This is consistent with our GSEA results as well. More importantly, we found that CCL5 is associated with immune-related molecules and pathways. We found that CCL5 status is associated with a variety of immune cells, including CD8 T cells (30), NK cells (31) and γδ T cells (32) that have been identified. Treg cells express the transcription factor Foxp3 and play a key role in maintaining immune homeostasis by inhibiting inflammatory responses in different biological environments (33). In most solid malignancies, high FOXP3 positive Treg infiltration in tumors is associated with poor prognosis (17); in contrast, patients with SCLC with FOXP3 positive levels have longer RFS (34). These immune-infiltrating cells are thought to promote the antitumor effects of the tumor microenvironment.

We also found statistical correlations in our data between CCL5 and immune checkpoints, including PD-1 and PD-L1 expressed in TILs. Surprisingly, patients with high CCL5 expression were predicted to respond better to immunotherapy. Thus, CCL5 may represent a potential predictor of immunotherapy. Based on the above analysis, we found a wide range of interactions between CCL5 and other immune biomarkers in SCLC.

The study has some limitations. First, the study included only three clinical cohorts. Only one cohort included immunotherapy patients. Our assumptions and results are based on a small sample size. Prospective and multicenter studies are needed in the future.

## Data Availability Statement

The original contributions presented in the study are included in the article/Supplementary Material, further inquiries can be directed to the corresponding author.

## Author Contributions

LG and YT: study design. YT, YH, and YN: data analysis, interpretation, and writing of the manuscript. LG, YT, and YN: revision of the manuscript. LS, YT, and YH: statistical analysis. All authors have reviewed the manuscript and approved the final version.

## Funding

This work was funded by the Science and Technology Planning Project of Guangdong Province (Grant No. 2019A030317020), the National Natural Science Foundation of China (Grant No. 81802254), and the Science and Technology Program of Guangzhou, China (No. 202002030359).

## Conflict of Interest

The authors declare that the research was conducted in the absence of any commercial or financial relationships that could be construed as a potential conflict of interest.

## Publisher's Note

All claims expressed in this article are solely those of the authors and do not necessarily represent those of their affiliated organizations, or those of the publisher, the editors and the reviewers. Any product that may be evaluated in this article, or claim that may be made by its manufacturer, is not guaranteed or endorsed by the publisher.

## Supplementary Material

Supplementary Figure 1 **(A)** The optimal cutoff point for the expression of CCL5 in the Jiang study. **(B)** The optimal cutoff point for the expression of CCL5 in the George study. **(C)** The optimal cutoff point for the expression of CCL5 in the Roper cohort.

Supplementary Table 1 The co-expression genes of CCL5.
