# Supplementary figures and images for: CCL5 as a Prognostic Marker for Survival and an Indicator for Immune Checkpoint Therapies in Small Cell Lung Cancer
Source: Front Med (Lausanne). 2022 Feb 17;9:834725. doi: 10.3389/fmed.2022.834725 (PMC8891515; doi:10.3389/fmed.2022.834725)

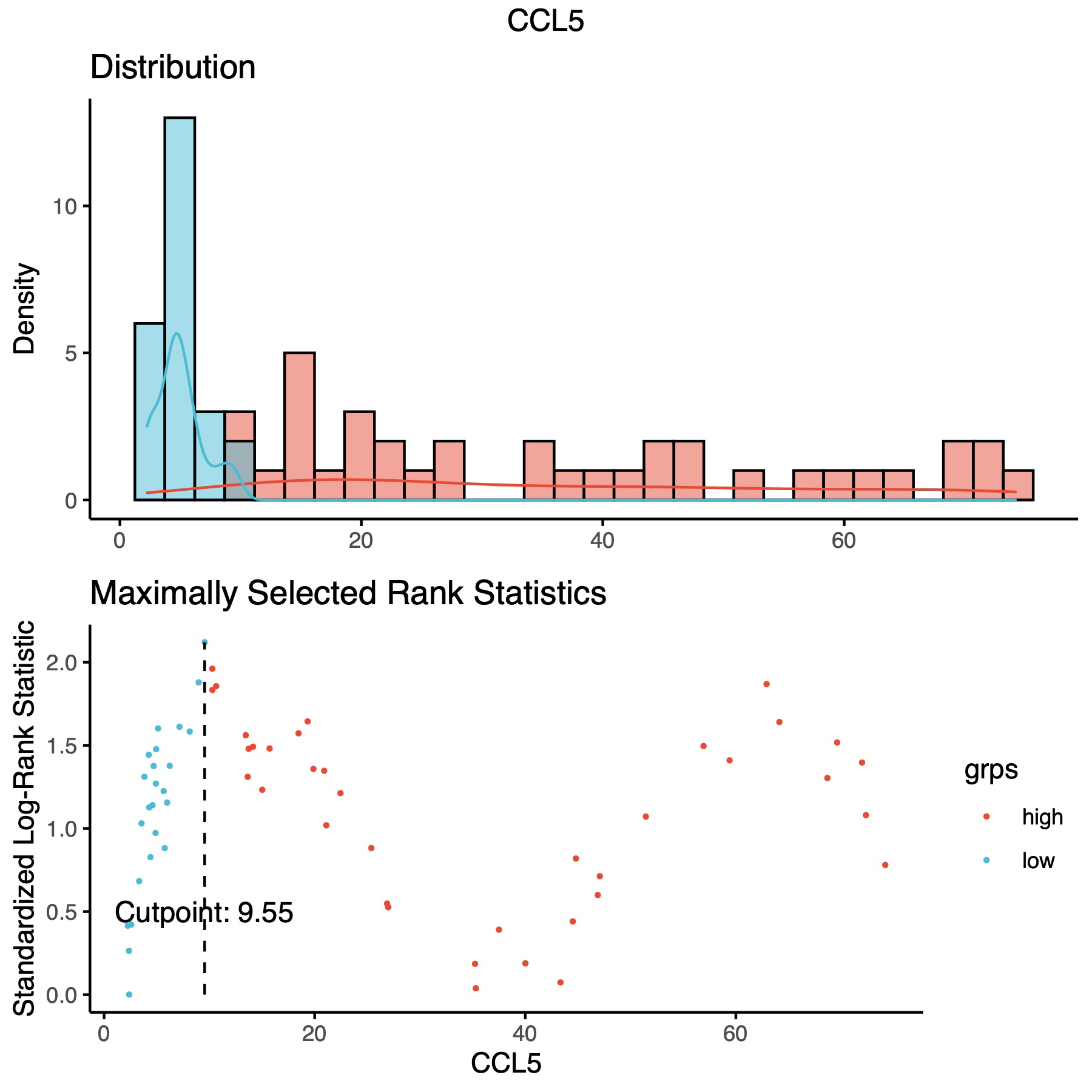

Supplement: Supplementary file 1 [file Image_2.JPEG]

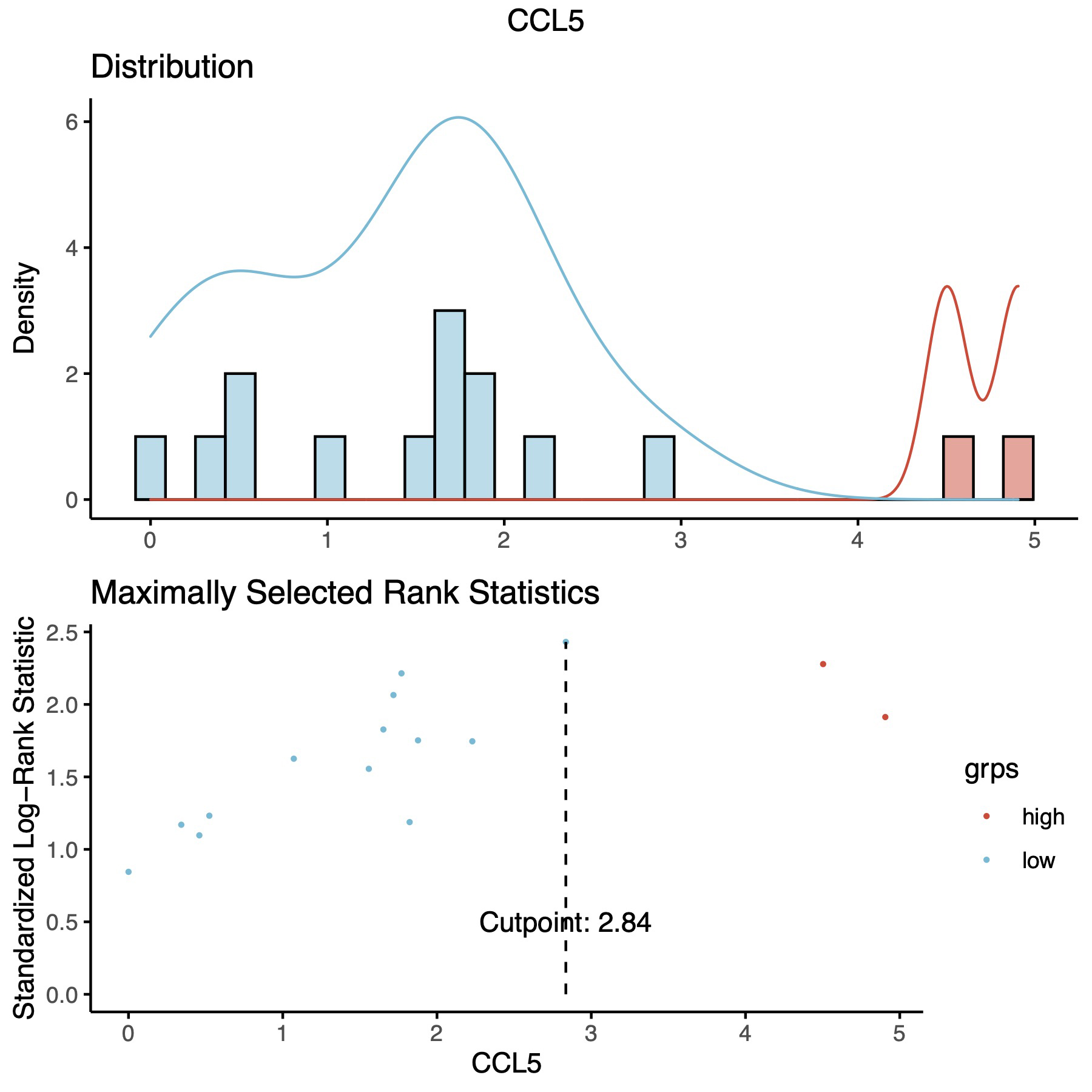

Supplement: Supplementary file 2 [file Image_3.JPEG]

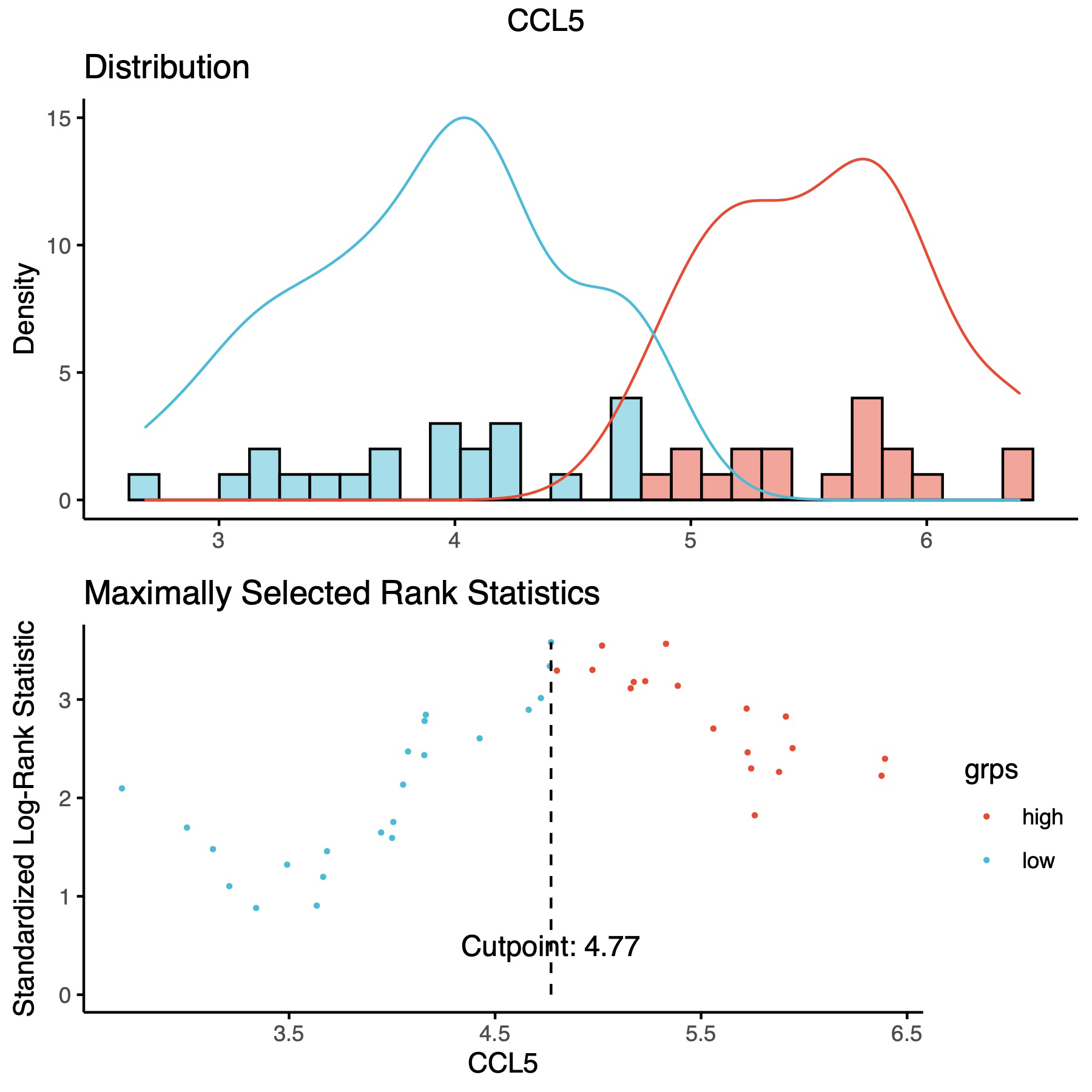

Supplement: Supplementary Figure 1 — (A) The optimal cutoff point for the expression of CCL5 in the Jiang study. (B) The optimal cutoff point for the expression of CCL5 in the George study. (C) The optimal cutoff point for the expression of CCL5 in the Roper cohort. [file Image_1.JPEG]
