# Supplementary material for: CCL5 as a Prognostic Marker for Survival and an Indicator for Immune Checkpoint Therapies in Small Cell Lung Cancer
Source: Front Med (Lausanne). 2022 Feb 17;9:834725. doi: 10.3389/fmed.2022.834725 (PMC8891515; doi:10.3389/fmed.2022.834725)
Supplement: Supplementary Table 1 — The co-expression genes of CCL5. [file Table_1.DOCX]

| **Genes** | **Pearson correlation coefficient** | ***p-Value*** |
| --- | --- | --- |
| CCL5 | 1 | 0 |
| LAPTM5 | 0.739900108 | 6.71E-15 |
| C3 | 0.737658577 | 8.91E-15 |
| HLA-DPB1 | 0.718063458 | 9.46E-14 |
| RAC2 | 0.691122304 | 1.79E-12 |
| JAK3 | 0.68154365 | 4.73E-12 |
| GPNMB | 0.679919976 | 5.56E-12 |
| SLCO2B1 | 0.669499644 | 1.52E-11 |
| MYO1F | 0.649047593 | 9.85E-11 |
| GBP4 | 0.64703925 | 1.17E-10 |
| RASAL3 | 0.645863099 | 1.30E-10 |
| SLA | 0.640869725 | 2.00E-10 |
| HLA-E | 0.635182049 | 3.23E-10 |
| TGM2 | 0.634194775 | 3.50E-10 |
| ITK | 0.632672597 | 3.98E-10 |
| ITGAL | 0.632219608 | 4.13E-10 |
| APOC1 | 0.630892633 | 4.60E-10 |
| CYTH4 | 0.630833395 | 4.63E-10 |
| FAM78A | 0.628617777 | 5.55E-10 |
| IL32 | 0.620598632 | 1.06E-09 |
| KLHL6 | 0.618681891 | 1.23E-09 |
| ITGB2 | 0.616035343 | 1.51E-09 |
| ARHGAP15 | 0.610954023 | 2.24E-09 |
| ARHGAP30 | 0.610807 | 2.27E-09 |
| ZAP70 | 0.610361312 | 2.34E-09 |
| FGD2 | 0.609497213 | 2.50E-09 |
| C1QC | 0.606925921 | 3.04E-09 |
| PTPRC | 0.60472692 | 3.59E-09 |
| CNN2 | 0.598641464 | 5.64E-09 |
| DOCK2 | 0.598240333 | 5.81E-09 |
| CD74 | 0.598072967 | 5.88E-09 |
| MVP | 0.595800087 | 6.94E-09 |
| MARCO | 0.595179888 | 7.26E-09 |
| CIITA | 0.593494686 | 8.20E-09 |
| HCLS1 | 0.592727152 | 8.67E-09 |
| CYR61 | 0.590939418 | 9.85E-09 |
| WIPF1 | 0.588569085 | 1.17E-08 |
| INPP5D | 0.586357513 | 1.36E-08 |
| CD3E | 0.584843848 | 1.52E-08 |
| CCDC69 | 0.584543474 | 1.55E-08 |
| MYO1G | 0.583040722 | 1.72E-08 |
| ICAM1 | 0.57966788 | 2.17E-08 |
| CTSH | 0.577148078 | 2.58E-08 |
| SASH3 | 0.573693348 | 3.26E-08 |
| PTGDS | 0.573031612 | 3.41E-08 |
| TNFRSF1B | 0.572732501 | 3.48E-08 |
| IL2RG | 0.572488099 | 3.54E-08 |
| HMHA1 | 0.571615498 | 3.75E-08 |
| LTBP2 | 0.571428883 | 3.80E-08 |
| RHOH | 0.571281814 | 3.84E-08 |
| NLRC5 | 0.567890242 | 4.81E-08 |
| LCP2 | 0.566760383 | 5.18E-08 |
| GNLY | 0.565458591 | 5.65E-08 |
| SERPING1 | 0.564785572 | 5.90E-08 |
| HLA-DRA | 0.564624428 | 5.96E-08 |
| CTSZ | 0.563856207 | 6.27E-08 |
| ITGAX | 0.563539923 | 6.40E-08 |
| KLF2 | 0.560355734 | 7.87E-08 |
| G0S2 | 0.56004429 | 8.03E-08 |
| AHR | 0.559082194 | 8.54E-08 |
| SRGN | 0.558921377 | 8.63E-08 |
| C1S | 0.558866057 | 8.66E-08 |
| MMP2 | 0.558835716 | 8.68E-08 |
| THBS1 | 0.558114635 | 9.09E-08 |
| TMEM119 | 0.554155229 | 1.17E-07 |
| HLA-DOA | 0.550960999 | 1.43E-07 |
| IL15RA | 0.550737236 | 1.45E-07 |
| SERPINB9 | 0.549910545 | 1.53E-07 |
| IL2RB | 0.54938496 | 1.58E-07 |
| NCKAP1L | 0.547993551 | 1.72E-07 |
| SELPLG | 0.547585002 | 1.76E-07 |
| LILRB4 | 0.547310939 | 1.79E-07 |
| LAIR1 | 0.545070763 | 2.06E-07 |
| TGFBR2 | 0.544468816 | 2.14E-07 |
| PRF1 | 0.542715442 | 2.38E-07 |
| TNFAIP3 | 0.542292803 | 2.44E-07 |
| HLA-DPA1 | 0.541877918 | 2.50E-07 |
| PLTP | 0.541243451 | 2.60E-07 |
| APBB1IP | 0.54120146 | 2.61E-07 |
| FYB | 0.540858702 | 2.66E-07 |
| APOL1 | 0.540345301 | 2.75E-07 |
| RARRES2 | 0.539900655 | 2.82E-07 |
| GBP5 | 0.539892985 | 2.82E-07 |
| SIGLEC1 | 0.538533754 | 3.07E-07 |
| CTSB | 0.538133182 | 3.14E-07 |
| CYTIP | 0.538098075 | 3.15E-07 |
| CXCL16 | 0.538090613 | 3.15E-07 |
| LSP1 | 0.533422622 | 4.16E-07 |
| NKG7 | 0.53263175 | 4.36E-07 |
| CYP1B1 | 0.531195271 | 4.74E-07 |
| LGALS9 | 0.530859452 | 4.84E-07 |
| IL7R | 0.530644051 | 4.90E-07 |
| TLR2 | 0.530232289 | 5.02E-07 |
| CYBB | 0.528681265 | 5.49E-07 |
| CXCL13 | 0.527910655 | 5.74E-07 |
| PLCB2 | 0.527893777 | 5.75E-07 |
| C1QA | 0.526616925 | 6.19E-07 |
| MS4A1 | 0.524635612 | 6.94E-07 |
| GIMAP5 | 0.52460657 | 6.95E-07 |
| TYROBP | 0.523140521 | 7.56E-07 |
| AMICA1 | 0.522709617 | 7.75E-07 |
| CD163 | 0.521750519 | 8.19E-07 |
| C1QB | 0.520873148 | 8.61E-07 |
| SLAMF1 | 0.519891505 | 9.10E-07 |
| CCR2 | 0.519644445 | 9.23E-07 |
| GIMAP2 | 0.519444936 | 9.33E-07 |
| NNMT | 0.51920107 | 9.46E-07 |
| CCR5 | 0.516978321 | 1.07E-06 |
| CLEC10A | 0.515957452 | 1.14E-06 |
| GBP1 | 0.513919933 | 1.27E-06 |
| CTSL1 | 0.511453972 | 1.46E-06 |
| GADD45B | 0.51088098 | 1.50E-06 |
| TYMP | 0.510838712 | 1.51E-06 |
| FGR | 0.509830383 | 1.59E-06 |
| IFITM3 | 0.509720132 | 1.60E-06 |
| BIN2 | 0.509496217 | 1.62E-06 |
| APOE | 0.508243558 | 1.74E-06 |
| IL10RA | 0.507447719 | 1.82E-06 |
| CCL4 | 0.506012156 | 1.96E-06 |
| LGALS1 | 0.505498697 | 2.02E-06 |
| SGK1 | 0.504782363 | 2.10E-06 |
| CD97 | 0.504542589 | 2.12E-06 |
| SLC2A3 | 0.504383876 | 2.14E-06 |
| CLEC2B | 0.504114594 | 2.17E-06 |
| CSF1R | 0.503557588 | 2.24E-06 |
| CCL21 | 0.502480521 | 2.37E-06 |
| CFI | 0.501233103 | 2.54E-06 |
| THBS2 | 0.501175393 | 2.55E-06 |
| GIMAP4 | 0.500828246 | 2.59E-06 |
| HLA-B | 0.500642325 | 2.62E-06 |
| CSF2RB | 0.499467047 | 2.79E-06 |
| IRF8 | 0.498132029 | 2.99E-06 |
| CTSC | 0.497574352 | 3.08E-06 |
| LRRK1 | 0.496869611 | 3.20E-06 |
| SLAMF7 | 0.496755422 | 3.22E-06 |
| CD2 | 0.496266567 | 3.30E-06 |
| TIMP1 | 0.49594495 | 3.36E-06 |
| MS4A6A | 0.495210852 | 3.49E-06 |
| TMC8 | 0.495056809 | 3.52E-06 |
| PMP22 | 0.494780237 | 3.57E-06 |
| CD79A | 0.49421168 | 3.68E-06 |
| GBP2 | 0.49354844 | 3.81E-06 |
| DPT | 0.492393407 | 4.04E-06 |
| HLA-C | 0.492256361 | 4.07E-06 |
| BST2 | 0.491007496 | 4.34E-06 |
| C7 | 0.490988663 | 4.35E-06 |
| EMILIN1 | 0.490353101 | 4.49E-06 |
| ISG20 | 0.48998693 | 4.58E-06 |
| AEBP1 | 0.489851975 | 4.61E-06 |
| MS4A7 | 0.489780484 | 4.63E-06 |
| PLA2G2D | 0.489360241 | 4.73E-06 |
| EMP1 | 0.489049218 | 4.80E-06 |
| SFRP2 | 0.488711364 | 4.89E-06 |
| LCP1 | 0.487827299 | 5.11E-06 |
| ALOX5AP | 0.487754057 | 5.13E-06 |
| PDGFRA | 0.487679374 | 5.15E-06 |
| GMFG | 0.487483507 | 5.20E-06 |
| CD69 | 0.487048504 | 5.32E-06 |
| SERPINE1 | 0.486923346 | 5.35E-06 |
| CCL19 | 0.48657858 | 5.45E-06 |
| CST7 | 0.485965754 | 5.62E-06 |
| AHNAK | 0.485743155 | 5.69E-06 |
| C1R | 0.485487347 | 5.76E-06 |
| SH2D1A | 0.485194949 | 5.85E-06 |
| CD44 | 0.484441675 | 6.07E-06 |
| RGS1 | 0.484240762 | 6.14E-06 |
| SOCS3 | 0.48348197 | 6.38E-06 |
| S100A4 | 0.482574063 | 6.67E-06 |
| CTGF | 0.482294406 | 6.77E-06 |
| SAMD9L | 0.482160458 | 6.82E-06 |
| C16orf54 | 0.482111435 | 6.83E-06 |
| CD53 | 0.481688966 | 6.98E-06 |
| A2M | 0.481198136 | 7.15E-06 |
| C10orf54 | 0.479713039 | 7.70E-06 |
| VIM | 0.479429482 | 7.81E-06 |
| IFITM2 | 0.477978073 | 8.40E-06 |
| CD38 | 0.477619958 | 8.55E-06 |
| ZFP36 | 0.476637895 | 8.97E-06 |
| BCL3 | 0.475851246 | 9.33E-06 |
| HLA-DMA | 0.475754903 | 9.37E-06 |
| LUM | 0.475603138 | 9.44E-06 |
| CFD | 0.475437222 | 9.52E-06 |
| IFI44L | 0.475427527 | 9.53E-06 |
| ZYX | 0.474869951 | 9.79E-06 |
| CTSS | 0.474786695 | 9.83E-06 |
| IQGAP2 | 0.474413995 | 1.00E-05 |
| SAMSN1 | 0.474219348 | 1.01E-05 |
| ZBED2 | 0.473529522 | 1.05E-05 |
| NCF4 | 0.47254145 | 1.10E-05 |
| CDH11 | 0.471441492 | 1.16E-05 |
| ABI3 | 0.471303623 | 1.17E-05 |
| PSTPIP1 | 0.470323522 | 1.22E-05 |
| TGFB1 | 0.469661457 | 1.26E-05 |
| ARHGAP18 | 0.468801167 | 1.32E-05 |
| APOL6 | 0.46801155 | 1.37E-05 |
| CTLA4 | 0.465969613 | 1.51E-05 |
| SERPINA1 | 0.465623662 | 1.53E-05 |
| CAPG | 0.465110287 | 1.57E-05 |
| GFPT2 | 0.464477567 | 1.62E-05 |
| FCER1G | 0.464429809 | 1.62E-05 |
| PTGER4 | 0.464313131 | 1.63E-05 |
| CTSE | 0.463835725 | 1.67E-05 |
| WAS | 0.462425382 | 1.78E-05 |
| HSH2D | 0.461405867 | 1.87E-05 |
| EMB | 0.461138838 | 1.89E-05 |
| BHLHE41 | 0.46073544 | 1.93E-05 |
| TNFRSF14 | 0.460271664 | 1.97E-05 |
| PDCD1 | 0.458081698 | 2.19E-05 |
| BATF2 | 0.457734097 | 2.22E-05 |
| FXYD5 | 0.457485751 | 2.25E-05 |
| RCSD1 | 0.457398977 | 2.26E-05 |
| POU2AF1 | 0.456419251 | 2.36E-05 |
| DAB2 | 0.456167539 | 2.39E-05 |
| ARHGDIB | 0.45484965 | 2.54E-05 |
| ZFP36L1 | 0.454062569 | 2.63E-05 |
| LYZ | 0.453870286 | 2.66E-05 |
| HAVCR2 | 0.453553838 | 2.70E-05 |
| P2RY8 | 0.452797965 | 2.79E-05 |
| SOD3 | 0.45143142 | 2.97E-05 |
| CFH | 0.450993422 | 3.03E-05 |
| CSDA | 0.450541013 | 3.09E-05 |
| HTRA3 | 0.450347698 | 3.12E-05 |
| CCR1 | 0.450343283 | 3.12E-05 |
| VAV1 | 0.449773191 | 3.20E-05 |
| C3AR1 | 0.44679344 | 3.67E-05 |
| MRC2 | 0.446604119 | 3.70E-05 |
| FPR1 | 0.445305933 | 3.92E-05 |
| C5AR1 | 0.444945434 | 3.98E-05 |
| GPRC5A | 0.444852071 | 4.00E-05 |
| SFTPA2 | 0.444198065 | 4.12E-05 |
| TAP1 | 0.442992115 | 4.35E-05 |
| CXCL9 | 0.442501679 | 4.44E-05 |
| CXCL12 | 0.442191026 | 4.50E-05 |
| CD247 | 0.441580677 | 4.63E-05 |
| CD3G | 0.441125711 | 4.72E-05 |
| RNASE1 | 0.440838126 | 4.78E-05 |
| CD48 | 0.440204118 | 4.92E-05 |
| DUSP1 | 0.4398744 | 4.99E-05 |
| LYN | 0.439689415 | 5.03E-05 |
| FBLN5 | 0.439427468 | 5.09E-05 |
| PIK3AP1 | 0.437877247 | 5.44E-05 |
| GBP3 | 0.437219446 | 5.60E-05 |
| HLA-DRB1 | 0.435424776 | 6.06E-05 |
| PLEK | 0.434867503 | 6.21E-05 |
| IGLL5 | 0.434278846 | 6.37E-05 |
| CD7 | 0.434142382 | 6.41E-05 |
| ODF3B | 0.432790368 | 6.79E-05 |
| MPEG1 | 0.432698511 | 6.82E-05 |
| CASP1 | 0.432587765 | 6.85E-05 |
| PLXDC2 | 0.431769019 | 7.10E-05 |
| SP140 | 0.431348707 | 7.22E-05 |
| MOXD1 | 0.431028938 | 7.32E-05 |
| IL27RA | 0.430884147 | 7.37E-05 |
| DCN | 0.430685892 | 7.43E-05 |
| SFRP4 | 0.43033163 | 7.55E-05 |
| PIM2 | 0.429923335 | 7.68E-05 |
| ITM2A | 0.428794948 | 8.06E-05 |
| GYPC | 0.428675287 | 8.10E-05 |
| TMEM150B | 0.426806667 | 8.77E-05 |
| FCGR3A | 0.426780874 | 8.77E-05 |
| BHLHE40 | 0.426531453 | 8.87E-05 |
| ARID5A | 0.425567144 | 9.24E-05 |
| C1orf38 | 0.425503314 | 9.26E-05 |
| ADH1B | 0.424770894 | 9.55E-05 |
| GNA15 | 0.424636953 | 9.60E-05 |
| NOTCH2NL | 0.424314789 | 9.73E-05 |
| DEF6 | 0.423924185 | 9.90E-05 |
| CCL2 | 0.422817397 | 0.000103641 |
| CCL3 | 0.420574871 | 0.000113774 |
| ABI3BP | 0.419247131 | 0.000120198 |
| VAMP5 | 0.418641711 | 0.000123236 |
| RNASE6 | 0.418464498 | 0.000124139 |
| FAM46C | 0.417798333 | 0.000127588 |
| NCF2 | 0.416790669 | 0.000132973 |
| ZFP36L2 | 0.416675911 | 0.0001336 |
| SLC2A5 | 0.415881403 | 0.000138012 |
| CD4 | 0.415477187 | 0.000140308 |
| IDO1 | 0.414540431 | 0.000145766 |
| FCGR2A | 0.414333484 | 0.000146998 |
| ISLR | 0.413917639 | 0.000149502 |
| RGS10 | 0.413154424 | 0.000154201 |
| PLEKHO2 | 0.410813541 | 0.000169476 |
| SLC34A2 | 0.410694074 | 0.000170292 |
| RARRES3 | 0.410179562 | 0.000173847 |
| DUSP5 | 0.409590735 | 0.000177999 |
| CD6 | 0.408762169 | 0.000183997 |
| RILPL2 | 0.408758069 | 0.000184027 |
| TPSAB1 | 0.408625192 | 0.000185006 |
| TLR8 | 0.407807472 | 0.000191138 |
| EPSTI1 | 0.406101107 | 0.000204541 |
| COL3A1 | 0.405866942 | 0.000206447 |
| VCAM1 | 0.403076864 | 0.000230443 |
| CCDC80 | 0.402449532 | 0.00023618 |
| CD300LF | 0.402245652 | 0.000238072 |
| MMP14 | 0.401735836 | 0.000242866 |
| FOLR2 | 0.401296176 | 0.000247071 |
| IFI16 | 0.401121839 | 0.000248757 |
| FMO2 | 0.400846877 | 0.000251438 |
| C8orf4 | 0.400268708 | 0.000257161 |
| HLA-DMB | 0.400259755 | 0.000257251 |
| PLCG2 | 0.400218266 | 0.000257666 |
| NCF1 | 0.399509654 | 0.000264857 |
| IL4I1 | 0.399438455 | 0.00026559 |
| CD300A | 0.399265036 | 0.000267382 |
| GIMAP6 | 0.398971678 | 0.000270439 |
| TNC | 0.398161861 | 0.000279045 |
| SFTPA1 | 0.397973595 | 0.000281082 |
| C10orf128 | 0.397350385 | 0.000287921 |
| LGALS2 | 0.3972778 | 0.000288728 |
| XAF1 | 0.39645944 | 0.000297964 |
| CD37 | 0.396429252 | 0.00029831 |
| C4BPA | 0.395831297 | 0.000305237 |
| AOAH | 0.395446102 | 0.000309777 |
| ACAP1 | 0.39532256 | 0.000311247 |
| RBM47 | 0.395076597 | 0.000314191 |
| PTPN7 | 0.394814273 | 0.000317359 |
| RUNDC3A | -0.394807096 | 0.000317446 |
| IL2RA | 0.389681906 | 0.000385549 |
| CHIT1 | 0.389491258 | 0.000388323 |
| RELB | 0.388513007 | 0.000402847 |
| TMEM37 | 0.388442921 | 0.000403907 |
| GM2A | 0.388312042 | 0.000405892 |
| CXCR3 | 0.386370766 | 0.000436406 |
| BLVRB | 0.386368512 | 0.000436443 |
| CCR7 | 0.386134019 | 0.000440268 |
| C11orf96 | 0.385835726 | 0.000445178 |
| MYC | 0.385671417 | 0.000447904 |
| RAB31 | 0.38303271 | 0.000493825 |
| HLA-DQA2 | 0.382932442 | 0.000495651 |
| LPXN | 0.38188684 | 0.000515074 |
| RNASET2 | 0.381640321 | 0.000519754 |
| CHI3L1 | 0.381434453 | 0.000523691 |
| TIFAB | 0.380855765 | 0.000534906 |
| CCL14 | 0.380652856 | 0.000538889 |
| S100A16 | 0.379639768 | 0.000559189 |
| MSR1 | 0.378828612 | 0.000575942 |
| DPYSL5 | -0.378813312 | 0.000576263 |
| SLC40A1 | 0.378602113 | 0.000580701 |
| BLNK | 0.37820538 | 0.000589124 |
| FCGR1A | 0.377630905 | 0.000601518 |
| MS4A4A | 0.376724879 | 0.00062155 |
| ATCAY | -0.376708365 | 0.00062192 |
| IGJ | 0.375411691 | 0.000651664 |
| GIMAP1 | 0.374650153 | 0.000669731 |
| CTSD | 0.372857279 | 0.000714082 |
| TMEM176B | 0.372338294 | 0.000727411 |
| SLPI | 0.370559674 | 0.000774826 |
| BTK | 0.368406125 | 0.000835993 |
| ELL2 | 0.367413511 | 0.000865637 |
| OAS2 | 0.36740823 | 0.000865797 |
| ALDH1A3 | 0.367374235 | 0.000866829 |
| TRADD | 0.366692267 | 0.000887773 |
| IL1R1 | 0.366445485 | 0.000895464 |
| GZMB | 0.365413666 | 0.000928282 |
| LILRB2 | 0.365010048 | 0.000941414 |
| INA | -0.362938824 | 0.001011495 |
| LY86 | 0.36164786 | 0.001057537 |
| GIMAP7 | 0.358993488 | 0.001158226 |
| JUNB | 0.35802149 | 0.001197217 |
| TNFSF10 | 0.357600722 | 0.001214461 |
| IER3 | 0.355538532 | 0.001302273 |
| MGP | 0.354877391 | 0.001331619 |
| IL18 | 0.349221748 | 0.001608159 |
| VSIG4 | 0.348303747 | 0.00165764 |
| FAM57B | -0.347834799 | 0.001683443 |
| KLF4 | 0.347691127 | 0.00169142 |
| SIRPA | 0.346040097 | 0.001785571 |
| MNDA | 0.344607207 | 0.001871081 |
| F13A1 | 0.342952387 | 0.001974412 |
| TIMP3 | 0.342060066 | 0.002032235 |
| HAPLN3 | 0.341931911 | 0.002040664 |
| SLC38A5 | 0.340705131 | 0.002122952 |
| DLL3 | -0.338779524 | 0.002258142 |
| ANXA1 | 0.33638076 | 0.002437339 |
| APLP1 | -0.33566965 | 0.002492859 |
| GIMAP8 | 0.335444273 | 0.002510691 |
| TSPO | 0.334759004 | 0.002565614 |
| UNC93B1 | 0.334741853 | 0.002567002 |
| EFEMP1 | 0.334458726 | 0.002590018 |
| SYT13 | -0.333939918 | 0.002632673 |
| PDCD1LG2 | 0.333333809 | 0.002683302 |
| TAGLN3 | -0.33303046 | 0.002708967 |
| BTN3A3 | 0.332574825 | 0.00274793 |
| MAST1 | -0.33252262 | 0.002752426 |
| CD14 | 0.33195742 | 0.002801527 |
| FCN1 | 0.331679327 | 0.002825973 |
| CYBA | 0.331606336 | 0.00283242 |
| RASSF4 | 0.331043761 | 0.00288256 |
| PDZK1IP1 | 0.330465298 | 0.002934942 |
| CARD11 | 0.329704477 | 0.003005133 |
| ADCY1 | -0.329586932 | 0.00301611 |
| DUSP2 | 0.329258746 | 0.003046949 |
| KYNU | 0.327803763 | 0.00318709 |
| DAPP1 | 0.327341193 | 0.003232836 |
| LTB | 0.326998599 | 0.003267093 |
| DUSP26 | -0.32663176 | 0.003304134 |
| FPR3 | 0.323453889 | 0.00364104 |
| DOK2 | 0.322312651 | 0.003769304 |
| GREM1 | 0.322214601 | 0.00378051 |
| CP | 0.32067718 | 0.003960134 |
| CD180 | 0.320647188 | 0.003963712 |
| DRAM1 | 0.315151381 | 0.004670091 |
| IL6 | 0.31192613 | 0.005134774 |
| CXCL14 | 0.310578296 | 0.005340801 |
| PLAU | 0.308240274 | 0.005715588 |
| DPYSL4 | -0.307927272 | 0.005767487 |
| GZMH | 0.299777602 | 0.007273813 |
| ADAMDEC1 | 0.298649134 | 0.007507568 |
| UBA7 | 0.296930764 | 0.007876205 |
| C1QL1 | -0.291509721 | 0.009145371 |
| ITGBL1 | 0.284436616 | 0.011068105 |
| CD40 | 0.280938257 | 0.012142921 |
| FERMT3 | 0.279813808 | 0.012507124 |
| GZMA | 0.279752554 | 0.012527233 |
| GLRX | 0.279081641 | 0.012749333 |
| GPX3 | 0.276329322 | 0.013696598 |
| COL10A1 | 0.270998072 | 0.015705823 |
| HLA-F | 0.270631272 | 0.015852957 |
| BATF | 0.269281676 | 0.016404591 |
| SIGLEC8 | 0.261533043 | 0.019902291 |
| FOXG1 | -0.260323085 | 0.020502349 |
| S1PR4 | 0.257592398 | 0.021913661 |
| MAN1A1 | 0.244800544 | 0.029678606 |
| JAKMIP2 | -0.237827451 | 0.034811197 |
| TDO2 | 0.235560442 | 0.036632108 |
| ITGB7 | 0.234296088 | 0.037681764 |
| TXNDC5 | 0.226293627 | 0.044922659 |
| L1CAM | -0.203311673 | 0.072321291 |
| PDZD4 | -0.190523813 | 0.092595059 |
| SCG3 | -0.180932688 | 0.110548506 |
| EMP3 | 0.173891961 | 0.125361483 |

*CCL5 as a Prognostic Marker for Survival and an Indicator for Immune Checkpoint Therapies in Small Cell lung Cancer*

Yichun Tang 1 †, Yueyang Hu2 †, Yuchun Niu1 †, Lei Sun, Linlang Guo1*

1.Department of Pathology, Zhujiang Hospital, Southern Medical University, Guangzhou, China

2. Department of Hepatobiliary Surgery, Zhujiang Hospital, Southern Medical University, Guangzhou, China.

 * Correspondence:

Linlang Guo

E-mail addresses: linlangg@yahoo.com (Linlang Guo)

Immunogenetics
